# *Mycobacterium avium* genotype is associated with the therapeutic response to lung infection

**DOI:** 10.1111/1469-0691.12285

**Published:** 2013-07-05

**Authors:** T Kikuchi, Y Kobashi, T Hirano, N Tode, A Santoso, T Tamada, S Fujimura, Y Mitsuhashi, Y Honda, T Nukiwa, M Kaku, A Watanabe, M Ichinose, M Drancourt

**Affiliations:** 1 Department of Respiratory Medicine, Tohoku University Graduate School of MedicineSendai, Japan; 2 Department of Respiratory Medicine, Tohoku University HospitalSendai, Japan; 3 Division of Respiratory Diseases, Department of Medicine, Kawasaki Medical SchoolKurashiki, Japan; 4 Department of Aging and Cancer, Research Division for Development of Anti-Infective Agents, Institute of Development, Tohoku UniversitySendai, Japan; 5 Department of Infection Control and Laboratory Diagnostics, Tohoku University Graduate School of MedicineSendai, Japan

**Keywords:** Logistic regression analysis, *Mycobacterium avium*, principal component analysis, therapeutic response, variable number tandem repeats

## Abstract

Factors that can interfere with the successful treatment of *Mycobacterium avium* lung infection have been inadequately studied. To identify a potent predictor of therapeutic responses of *M. avium* lung infection, we analyzed variable number tandem repeats (VNTR) at 16 minisatellite loci of *M. avium* clinical isolates. Associations between the VNTR profiling data and a therapeutic response were evaluated in 59 subjects with *M. avium* lung infection. *M. avium* lung infection of 30 subjects in whom clarithromycin-containing regimens produced microbiological and radiographic improvement was defined as responsive disease, while that of the remaining 29 subjects was defined as refractory disease. In phylogenetic analysis using the genotypic distance aggregated from 16-dimensional VNTR data, 59 *M. avium* isolates were divided into three clusters, which showed a nearly significant association with therapeutic responses (p 0.06). We then subjected the raw 16-dimensional VNTR data directly to principal component analysis, and identified the genetic features that were significantly associated with the therapeutic response (p <0.05). By further analysis of logistic regression with a stepwise variable-selection, we constructed the highest likelihood multivariate model, adjusted for age, to predict a therapeutic response, using VNTR data from only four minisatellite loci. In conclusion, we identified four mycobacterial minisatellite loci that together were associated with the therapeutic response of *M. avium* lung infections.

## Introduction

*Mycobacterium avium* is the pathogen that accounts for most cases of non-tuberculous mycobacterial lung infection, and an increase in its prevalence worldwide has been noted 1–5. In the treatment of *M. avium* lung infections, aggressive macrolide-based multidrug therapy for 12–24 months is recommended 1,2,5. However, the lack of factors predictive of the therapeutic response, with the exception of macrolide resistance predicting treatment failure, often discourages clinicians from starting the long-term, aggressive therapy required 1,2,5,6. This is particularly problematic when the symptoms of the patient are so moderate that treatment-related adverse effects may be worse than the lung disease itself 2,7.

Methods for molecular typing of mycobacteria have been developed, especially for investigation of *Mycobacterium tuberculosis* epidemiology 2,8,9. Among them, evaluation of variable number tandem repeats (VNTR) by polymerase chain reaction (PCR) has received particular attention, due to both its discriminatory ability and its convenience 10,11. Its discriminatory power depends on the number of evaluated minisatellite loci, of which 12–24 have been evaluated for mycobacterial typing 10. Each mycobacterial isolate is profiled as a set of 12–24 integer values that correspond to the numbers of repeats at the evaluated minisatellite loci. To date, the raw digital data of the VNTR profiles have been analyzed after analog interpretation in a phylogenetic tree 9,12,13. In the present study, our findings demonstrate that the 16-digit VNTR data, and particularly those from the four selected minisatellite loci, may be directly analyzed, without the digital-to-analog conversion required for phylogenetic analysis, for use as predictors of the therapeutic response in *M. avium* lung infection.

## Material and Methods

### Study population

Our analysis included 59 subjects who were diagnosed with *M. avium* lung infection according to the official American Thoracic Society/Infectious Diseases Society of America (ATS/IDSA) statement, and were treated with clarithromycin-based multidrug regimens at the Tohoku University Hospital or at the Kawasaki Medical School Hospital 2. Patients who had completed at least 6 months of therapy were chosen for this study. Among them, 17 subjects from the Tohoku University Hospital had been included in a previous unrelated study 13. Therapeutic response was evaluated 6 months after the start of medication, based on microbiological and radiographic findings. Microbiological improvement was determined as sputum culture negativity for 3 months at 4–6 months after the start of treatment; radiographic regression after 6 months of treatment was determined on the basis of standards described previously 14. Subjects who met both microbiological and radiographic criteria were classified as having responsive *M. avium* lung disease, while those whose results failed to meet either of the criteria were classified as having refractory disease. The Institutional Review Board at Tohoku University Graduate School of Medicine (Sendai, Japan) approved the study.

### VNTR profiling

Details of the mycobacterial genotyping methods used in the VNTR analysis have been described previously 13. Briefly, minisatellite loci were amplified using PCR in *M. avium* respiratory isolates (i.e. from sputa or bronchial washes) of enrolled subjects before starting the anti-mycobacterial treatment. After electrophoretic separation on a 2.5% agarose gel, the sizes of PCR amplicons were estimated using the ChemiDoc XRS system (Bio-Rad Laboratories, Hercules, CA, USA). The number of repeat units was determined at 16 minisatellite loci and considered to be the VNTR profile of each *M. avium* isolate.

### Phylogenetic analysis

The genotypic diversity of *M. avium* clinical isolates in a phylogenetic tree was assessed from the VNTR profiles, as described previously 13. Briefly, an unrooted dendrogram was built based on Manhattan distances between each pair of *M. avium* isolates, using the neighbour-joining algorithm contained in the PHYLIP software, version 3.67. Manhattan distance was calculated by the following formula:
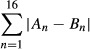
where *An* and *Bn* are the number of repeat units in the *n*th minisatellite locus of *M. avium* isolates A and B, respectively.

### Principal component analysis (PCA)

To discriminate genetic groups of *M. avium* isolates directly using VNTR components, PCA was used. Raw numbers of tandem repeats at all 16 minisatellite loci for 59 *M. avium* isolates were utilized to determine principal components using the JMP Pro software, version 9.0.2 (SAS Institute, Cary, NC, USA). Individual *M. avium* isolates were depicted on a scatter plot of the two principal components that accounted for the two greatest degrees of variance.

### Statistical analysis

Normally distributed continuous data, non-normally distributed data and categorical data were evaluated between the two study groups using Student's *t*-tests, Mann–Whitney tests and Fisher's exact tests, respectively. Numbers of tandem repeats at each minisatellite locus were compared between subjects with responsive and refractory *M. avium* lung disease, on the basis of a logistic regression model adjusted for age. The result of the model was reported as an age-adjusted odds ratio for responsive vs. refractory disease. The likelihood of the model was examined using the chi-square test to evaluate the null hypothesis that all variable coefficients were zero (i.e. no variables have any effect on the model). The probability of successful treatment was calculated using the age-adjusted multivariate logistic-regression model with VNTR profile data at MATR-2, -3, -8 and -16 loci that had been selected with the use of a stepwise variable-selection procedure. p values of <0.05 were considered to indicate statistical significance. All analyses were performed using the JMP Pro software, version 9.0.2 (SAS Institute).

## Results

### Characterization of subjects

The subject group with responsive *M. avium* lung disease included 10 males and 20 females with a mean age of 61 ± 12 years at the start of treatment (Table1). The subject group with refractory disease included 29 persons who were similar for age (64 ± 11 years, p 0.41), sex (eight males and 21 females, p 0.78) and pulmonary co-morbidity (refractory group, two subjects with chronic obstructive pulmonary disease and one with bronchiectasis; responsive group, one subject with lung cancer and one with bronchial asthma; p 0.67). There were no clearly significant differences in other radiographic and microbiological characteristics between the responsive and refractory subjects, although subjects with responsive disease tended to have a higher proportion of nodular bronchiectatic features (80% vs. 76%, p 0.76), to have fewer lung segments affected on chest computed tomography scans (6.7 vs. 7.7, p 0.2) and to have *M. avium* isolates more susceptible to clarithromycin (MIC_50_, 1 vs. 1 mg/L; MIC_80_, 2 vs. 4 mg/L; p 0.63). No clarithromycin-resistant *M. avium* strains (MIC, 32 mg/L or greater 2) were included in either subject group. As for the treatment strategy, no significant differences were observed between the two groups of subjects (Table S1).

**Table 1:** Demographic and clinical characteristics of subjects with *Mycobacterium avium* lung infection

Characteristic	Responsive disease^a^ (*n* = 30)	Refractory disease (*n* = 29)	p
Age at start of treatment^b^	61 ± 12	64 ± 11	0.41
Male sex, *n* (%)	10 (33)	8 (28)	0.78
History of lung disease, *n* (%)	2 (7)	3 (10)	0.67
Clinical feature, *n* (%)			0.76
Fibrocavitary	6 (20)	7 (24)	
Nodular bronchiectatic	24 (80)	22 (76)	
Affected segment, *n*^b^	6.7 ± 3.0	7.7 ± 3.1	0.2
Sensitivity to clarithromycin^c^			0.63
MIC_50_ (mg/L)	1	1	
MIC_80_ (mg/L)	2	4	
VNTR cluster, *n* (%)^d^			0.06
Cluster A	10 (33)	3 (10)	
Cluster B	9 (30)	8 (28)	
Cluster C	11 (37)	18 (62)	

a Responsive disease was defined as both microbiological and radiographic improvement.

b Values represent the means ± standard deviations.

c MIC_50_ and MIC_80_ refer to the minimum concentrations of clarithromycin required to inhibit growth of 50 and 80% of *M. avium* isolates, respectively.

d VNTR cluster is defined in Fig. 2.

### Genotyping and phylogenetic analysis of *M. avium* isolates

**Fig 1:**
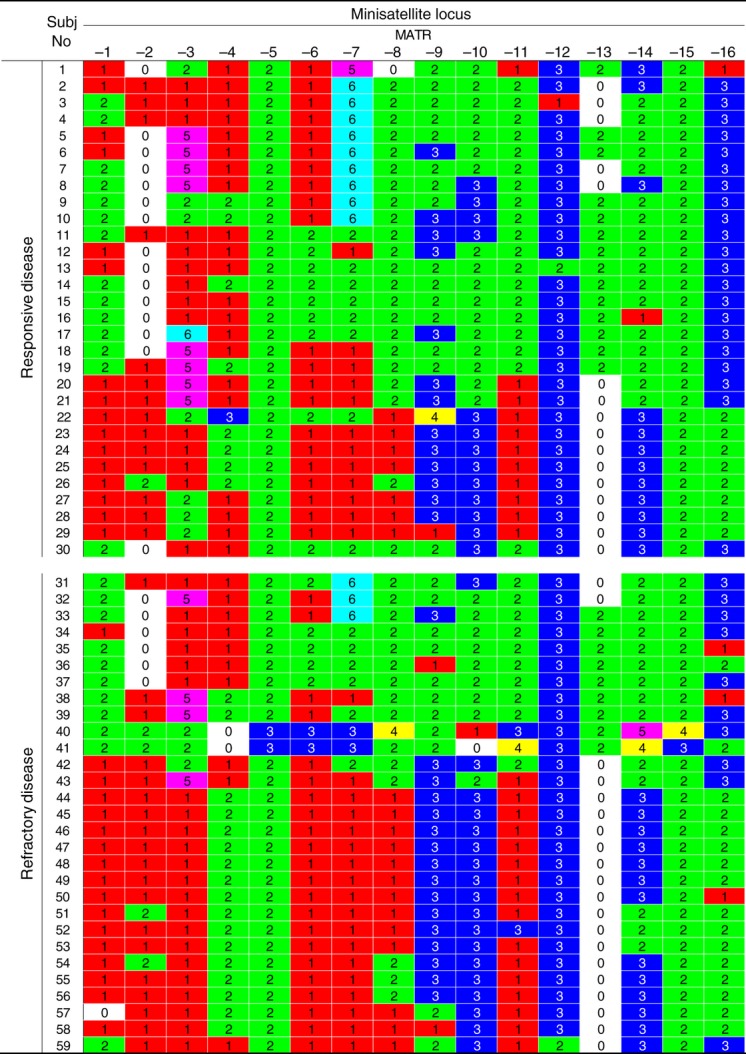
VNTR profiles of clinical *Mycobacterium avium* isolates. *M. avium* genomic DNA was isolated from 59 subjects: subjects 1–30 with *M. avium* lung disease responsive to clarithromycin-based multidrug regimens, and subjects 31–59 with lung disease refractory to treatment. The minisatellite loci, MATR-1 to -16, were amplified from mycobacterial DNA by PCR. From the size of the PCR product, the number of repeat units in each minisatellite locus was calculated. The numbers of tandem repeat units at 16 minisatellite loci are shown for each *M. avium* isolate. Boxes of the same colour represent the same number of tandem repeats.

To evaluate the association of mycobacterial genotype with therapeutic response, we obtained the VNTR profiling data of all *M. avium* isolates, determining the numbers of tandemly repeated motifs at minisatellite loci in the genome (Fig.1). The VNTR profile of *M. avium* isolated from each subject consists of 16 integer values from 16 minisatellite loci, MATR-1 to -16; subjects 1–30 had responsive *M. avium* lung disease, while subjects 31–59 had refractory disease. The 16 × 59 seemingly unrelated data points permit further evaluation of the association between mycobacterial genotype and the clinical response to anti-mycobacterial treatment.

**Fig 2:**
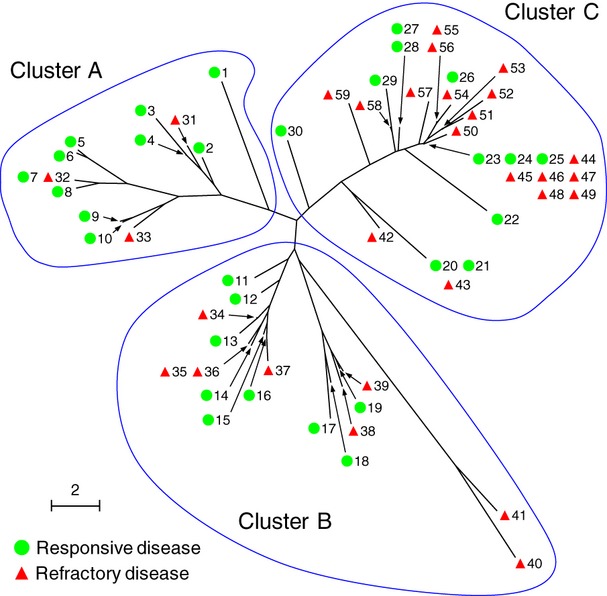
Phylogenetic analysis of *Mycobacterium avium* isolates from 59 subjects with lung infections. From all VNTR profile data shown in Fig.1, the Manhattan distance between each pair of isolates from subjects with responsive (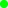) or refractory disease (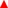) was calculated. The phylogenetic distribution was analysed by a neighbour-joining algorithm, and is shown together with subject numbers as a radial dendrogram. The three major branches of *M. avium* are designated clusters (a), (b) and (c). The scale bar indicates genetic distance.

When calculating genotypic diversity in the VNTR profiles as the Manhattan distance between each pair of *M. avium* isolates, we found phylogenetic lineages to be associated with the therapeutic response (Fig.2). In the phylogenetic tree, *M. avium* clinical isolates were classified into three major clusters, designated as A, B and C. The classification yields a nearly significant association with the therapeutic response; clusters A and C were more likely to include *M. avium* isolates from responsive and refractory lung disease, respectively, while cluster B did not appear to have such a propensity (responsive/refractory disease: cluster A, 10/3; cluster B, 9/8; cluster C, 11/18; p 0.06; Table1). These data suggest that the phylogenetic distribution of *M. avium* isolates accounts, at least in part, for the clinical differences in the therapeutic responses of the lung infections.

### Principal component analysis (PCA) of 16-digit VNTR profiling data

**Fig 3:**
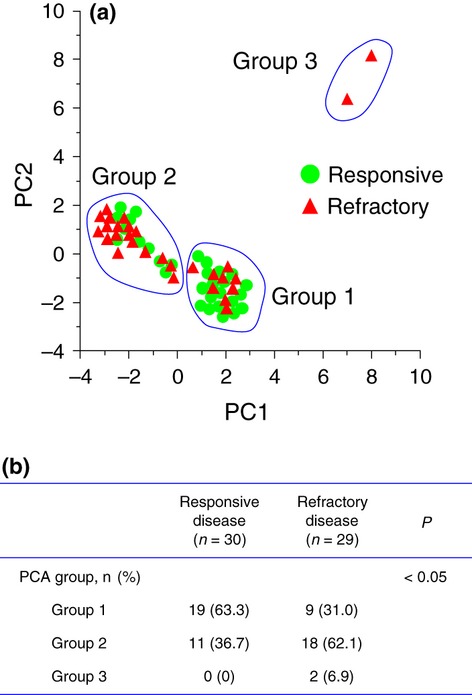
Genetic groups discriminated by VNTR profile components. (a) Individual *Mycobacterium avium* isolates represented in the principal component analysis (PCA) space. PCA scores for numbers of tandem repeats at 16 minisatellite loci were plotted as a scatter of *M. avium* isolates from 59 subjects with responsive (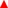) or refractory disease (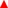). PC1 and PC2 are the first two principal components and explain 55.7% of the variance (36% and 19.7% for PC1 and PC2, respectively). The scatter plots revealed three genetic groups, which are designated Groups 1, 2 and 3. (b). Proportions of the genetic groups among subjects with responsive or refractory disease. p values for associations of genetic groups with therapeutic response were calculated using Fisher's exact test.

To confirm that the diversity of mycobacterial VNTR profiles reflected the therapeutic response, we carried out PCA using 16-digit VNTR data, without calculating Manhattan distances (Fig.3). Because the Manhattan distance is defined as the sum of the absolute differences in each dimension, the aggregate measurements seem less sensitive to genetic variability. In accordance with this view, scatter plots of principal component (PC) 1 and PC2 from PCA of the raw VNTR data as 16-digit representation revealed that *M. avium* clinical isolates were well resolved into three major groups, designated Groups 1, 2 and 3 to avoid confusion with the clustering shown in Fig.2 (Fig.** **3a). In this analysis, PC1 and PC2 accounted for 36% and 19.7% of the total genetic variation, respectively, and these two components together for 55.7% of the total variation. The amount of variation explained by PC3 was small (<8%), and therefore, additional components from PC3 onward were disregarded.

Inspection of clinical information linked to *M. avium* isolates allowed PCA to detect a correlation between the therapeutic response and the scattering pattern of *M. avium* in the PCA space (Fig.3b). *M. avium* isolates from responsive lung disease tended to form Group 1, which comprises medium PC1 scores, 0 through to 3 (19 of 30, 63.3%). In contrast, those with refractory disease tended to form Group 2, which comprises low PC1 scores, −4 through to 0 (18 of 29, 62.1%). Two *M. avium* isolates, both of which were cultured from subjects with refractory lung disease (i.e. subjects 40 and 41), were assigned to Group 3, defined by high PC1 and PC2 scores (PC1/PC2 score: subject 40, 8/8.2; subject 41, 7/6.4). The PCA distribution of *M. avium* isolates was significantly associated with the therapeutic response (p <0.05, Fig.3b). These observations support analysis of the raw 16-digit VNTR data of *M. avium* to predict the therapeutic responses of lung infections.

### Prediction of the therapeutic response with VNTR data from minimal loci

Using the raw 16-digit VNTR data of *M. avium*, we employed logistic regression analysis to predict the likelihood of successful treatment of *M. avium* lung infection (Table2). When the raw VNTR data at each minisatellite loci were independently tested in terms of their association with responsive *M. avium* lung disease, the number of repeat units at three minisatellite loci, MATR-2, -7 and -16, showed significant associations in a logistic regression analysis after adjustment for age. *M. avium* exhibiting fewer repeat units at MATR-2 and more repeat units at MATR-7 and MATR-16, were significantly more likely to cause lung disease responsive to anti-mycobacterial therapy (age-adjusted odds ratio for responsive disease vs. refractory disease (95% confidence interval): MATR-2, 0.27 (0.09–0.69), p <0.05; MATR-7, 1.35 (1.02–1.87), p <0.05; MATR-16, 3.45 (1.38–9.79), p <0.05; Table2). Of these, two models using VNTR data at MATR-2 and MATR-16 loci were sufficient to predict the probability of successful treatment (MATR-2, χ^2^ = 8.4, p <0.05; MATR-16, χ^2^ = 7.95, p <0.05; Table2).

**Table 2:** Age-adjusted odds ratios and 95% confidence intervals for the number of repeat units at each minisatellite locus in subjects with responsive vs. refractory disease^a^

Minisatellite locus	Odds ratio (95% CI)^b^	Chi-square	p	Model test^c^	
				Chi-square	p
MATR-1	1.59 (0.59–4.42)	0.83	0.36	1.55	0.46
MATR-2	0.27 (0.09–0.69)	6.52	<0.05	8.4	<0.05
MATR-3	1.39 (1–2.02)	3.49	0.06	4.56	0.1
MATR-4	0.54 (0.21–1.33)	1.72	0.19	2.49	0.29
MATR-5	0 (0)	0	1	3.48	0.18
MATR-6	0.99 (0.37–2.68)	0	0.99	0.71	0.7
MATR-7	1.35 (1.02–1.87)	3.97	<0.05	5.17	0.08
MATR-8	1.24 (0.52–3.09)	0.23	0.63	0.95	0.62
MATR-9	0.89 (0.37–2.08)	0.07	0.79	0.79	0.68
MATR-10	0.73 (0.29–1.71)	0.51	0.47	1.24	0.54
MATR-11	1.21 (0.53–2.84)	0.21	0.65	0.92	0.63
MATR-12	0.44 (0.02–2.58)	0.62	0.43	1.47	0.48
MATR-13	1.46 (0.85–2.55)	1.82	0.18	2.58	0.27
MATR-14	0.52 (0.2–1.18)	2.19	0.14	3.12	0.21
MATR-15	0 (0)	0	1	3.48	0.18
MATR-16	3.45 (1.38–9.79)	6.24	<0.05	7.95	<0.05

a Age-adjusted odds ratios were generated using a logistic regression model.

b CI denotes confidence interval.

c A chi-square test was used to examine whether the variable had an effect on the model.

To improve the ability of this univariate model to estimate the probability that patients would show a favourable response to anti-mycobacterial medication, we conducted a multivariate logistic regression analysis of VNTR data at minisatellite loci. The VNTR data at 16 minisatellite loci were included in a stepwise variable-selection analysis, and four loci, MATR-2, -3, -8 and -16, appeared to have a cumulative association with responsive *M. avium* lung disease after adjustment for age. The final model in which these four minisatellite loci were retained remained significant, and its proficiency was further improved relative to the univariate models using MATR-2 or MATR-16 (χ^2^ = 15.14, p <0.01). Additional adjustment for the clarithromycin susceptibility resulted in a small improvement of this multivariate model (χ^2^ = 15.17, p 0.02).

## Discussion

The present study was based on the assumption that some mycobacterial factors of *M. avium* are of therapeutic relevance in lung infections. Our data support this idea. In the study cohort, which comprised 59 subjects with *M. avium* lung infection, therapeutic response had a nearly significant association with the phylogenetic distribution of the *M. avium* isolates, when this was assessed by the Manhattan distance aggregated over VNTR data from 16 minisatellite loci. The association was further clarified by applying the raw VNTR data to principal component analysis without distance aggregation. In particular, the raw VNTR data from four selected minisatellite loci facilitated development of a significant multivariate logistic regression model to predict the therapeutic response.

Many questions regarding clinical management of *M. avium* lung infection remain unanswered 1,2,5. The most crucial question that has been discussed is that of the optimal therapy 1,2,5. Although combination antibiotic therapy containing clarithromycin or azithromycin offers the best chance of treatment response, the current regimens have major limitations. Even long-duration multidrug therapy, with its frequent intolerable adverse drug reactions, leads to relatively high failure rates, ranging from 20 to 70% 1,2,5. Thus, many clinicians hesitate to initiate potentially toxic treatment against *M. avium* lung infection in the absence of a method of predicting the therapeutic response. Particularly when the disease presents in an indolent fashion, drug-related adverse events may be more deleterious than the disease process itself. Against this background, the present study highlights clinical factors that enable prediction of the therapeutic response to standard clarithromycin-containing regimens.

Although still insufficient, our understanding of the pathophysiology of *M. avium* lung disease has increased with regard to both host conditions and the mycobacterial factors emphasized in this study 1,2,5,15,16. Five major conditions have been recognized as predisposing host factors for non-tuberculous mycobacterial diseases: (i) human immunodeficiency virus infection 17, (ii) genetic mutations in signalling pathways of interferon-γ and interleukin-12 18,19, (iii) structural lung disease such as chronic obstructive pulmonary disease, bronchiectasis, cystic fibrosis, pneumoconiosis, prior tuberculosis, pulmonary alveolar proteinosis and oesophageal motility disorders 20, (iv) body morphotype, such as scoliosis, pectus excavatum, mitral valve prolapse and joint hypermobility 21, and (v) genetic HLA, SLC11A1 (NRAMP1), MICA and TLR2 polymorphisms 22–28. Despite the above, there is no clear explanation of why some individuals, especially elderly women, become ill with poor or good prognoses 2,29. Recently, relevant studies have shown that factors related to overall health status, such as body mass index and anaemia, affect all-cause mortality of patients with *M. avium* lung disease, but that the therapeutic regimen employed as the first line (e.g. no therapy or combination therapy with one to five drugs) does not 30. Thus, it seems probable that our age-adjusted model to predict the therapeutic response of *M. avium* lung infection may be further improved by considering host factors indicative of the underlying overall health status of the patient.

Collectively, out data indicate that the therapeutic response of *M. avium* lung infection is associated with mycobacterial grouping based on VNTR. Genotypic characterization of *M. avium* isolates may be a useful strategy for predicting the clinical outcome of medication.

## Supporting Information

Supplementary **Table S1.** Details of the treatment for subjects with *M. avium* lung infection.
