# Supplementary material for: Mycobacterium avium genotype is associated with the therapeutic response to lung infection
Source: Clin Microbiol Infect. 2013 Jul 5;20(3):256–62. doi: 10.1111/1469-0691.12285 (PMC4231998; doi:10.1111/1469-0691.12285)
Supplement: Supplementary file 1 — Supplementary Table S1. Details of the treatment for subjects with M. avium lung infection. [file clm0020-0256-SD1.pdf]

Table S1. Details of the treatment for subjects with *M. avium* lung infection.

Detail	Responsive disease (n = 30)	Refractory disease (n = 29)	*P*
Duration (month)*	15 ± 5.4	14 ± 6.6	0.6
Used drug, n*	3.5 ± 0.8	3.4 ± 0.9	0.71
Regimen†			0.75
CAM+RFP+EB+SM/FQ	20	17	
CAM+RFP+EB	7	7	
CAM+other combinations	3	5	
Dose of clarithromycin (mg)*	680 ± 179	648 ± 190	0.47

*Values represent the means ± standard deviations.

†CAM, clarithromycin; RFP, rifampicin; EB, ethambutol; SM/FQ, streptomycin and/or fluoroquinolone including levofloxacin, moxifloxacin, and gatifloxacin.
